# Influence of Surface Chemistry on the Electrochemical Performance of Biomass-Derived Carbon Electrodes for its Use as Supercapacitors

**DOI:** 10.3390/ma12152458

**Published:** 2019-08-02

**Authors:** Abdelhakim Elmouwahidi, Esther Bailón-García, Luis A. Romero-Cano, Ana I. Zárate-Guzmán, Agustín F. Pérez-Cadenas, Francisco Carrasco-Marín

**Affiliations:** 1 Research Group in Carbon Materials, Inorganic Chemistry Department, Faculty of Sciences, University of Granada, Campus Fuente Nueva s/n. 18071 Granada, Spain; 2 Facultad de Ciencias Químicas, Universidad Autónoma de Guadalajara, Av. Patria 1201, Zapopan, Jalisco C. P. 45129, Mexico; 3 Centro de Investigación y Desarrollo Tecnológico en Electroquímica (CIDETEQ) S.C., Parque Tecnológico Sanfandila, Pedro Escobedo, Querétaro 760703, Mexico

**Keywords:** nitrogen and oxygen doped activated carbon, surface chemistry, supercapacitor capacitance, energy power density

## Abstract

Activated carbons prepared by chemical activation from three different types of waste woods were treated with four agents: melamine, ammonium carbamate, nitric acid, and ammonium persulfate, for the introduction of nitrogen and oxygen groups on the surface of materials. The results indicate that the presence of the heteroatoms enhances the capacitance, energy density, and power density of all samples. The samples treated with ammonium persulfate show the maximum of capacitance of 290 F g^−1^ while for the melamine, ammonium carbamate, and nitric acid treatments, the samples reached the maximum capacitances values of 283, 280, and 455 F g^−1^ respectively. This remarkable electro-chemical performance, as the high specific capacitances can be due to several reasons: i) The excellent and adequate textural characteristics makes possible a large adsorption interface for electrolyte to form the electrical double layer, leading to a great electrochemical double layer capacitance. ii) The doping with hetero-atoms enhances the surface interaction of these materials with the aqueous electrolyte, increasing the accessibility of electrolyte ions. iii) The hetero-atoms groups can also provide considerable pseudo-capacitance improving the overall capacitance.

## 1. Introduction

As a consequence of the change in the energy model to which we are involved, together with the challenge of mitigating climate change, one of the main technologies with serious possibilities of being implemented are high-performance energy storage and conversion devices as fuel cells, batteries or supercapacitors [1,2,3]. Supercapacitors are electrochemical devices capable of provide an unusually high energy amount, that is high power density. During the rechargeable electrochemical cycles the charge carriers migrate reciprocally between electrolytes and electrodes. Supercapacitors have also excellent reversibility together with long cycle life, therefore supercapacitors are specifically good candidates for large-scale applications of portable and automotive electronic systems. Nevertheless, one inconvenience of supercapacitors is the relatively low energy density, an in this line, many efforts are being made by designing and optimizing the materials for the corresponding electrodes.

Carbon based materials are probably the best candidates for this type of electrochemical applications taking in account the extended literature published during the last years [4,5,6]. On the other hand, a different type of precursors are used for the preparation of activated carbon from waste agriculture products such as olive stone [7,8,9], melia azedarach stones [10], argan seed shells [11], coconut [12,13], etc., by using different methods of chemical and physical activation [14,15]. The use of biomass wastes for carbon electrodes preparation and its use as supercapacitors is one of the best available options, not only for their very high electro-chemical performance but also for the economy of the synthesis process [3]. Indeed, for the electrochemical applications, the pore structure and an adequate surface chemistry are crucial, and carbon-based materials obtained from woods are easy tunable in both ways. For example, the capacitive behavior of carbon materials can be further improved by the presence of active species that contribute to the total specific capacitance by the pseudo-capacitive effect [16,17]. Others works have found that functional groups containing heteroatoms such as O and N are very favourable to improve the capacitance, and these groups can be introduced using different doping methods, as chemical oxidations, plasma treatment, or electrochemical treatments. [18,19,20]. The main aim of oxidation of a carbon surface is obtaining a more hydrophilic surface structure with groups such as carboxyl groups [21,22]. When oxygen containing groups are present on the surface of the activated carbon they affect the capacitance of this material mainly enhancing its wettability, and therefore increasing the capacitance and providing high energy and power densities; these type of groups can also produce pseudo capacitance effects. Several types of compounds have been used as oxidizers: ammonium per sulfate, sodium hypochlorite and permanganate, concentrated nitric or sulfuric acid or hydrogen peroxide. Furthermore, it has been reported that nitrogen groups modify the electron donor/acceptor characteristics of carbon materials depending on the type of interactions between the nitrogen and carbon atoms. The nitrogen-containing groups generally provide basic property, which could improve the interaction between carbon materials and acid molecules, such as dipole-dipole, H-bonding, covalent bonding, among others. The nitrogen groups can be formed by treatment with urea, melamine, nitric acid, and others types of containing nitrogen molecules [16,17,23].

In this work we present the treatment of three types of activated carbons, produced from chemical activation of waste woods, with four agents: nitric acid and ammonium persulfate for the introduction of oxygen groups; and melamine and ammonium carbamate for the introduction of nitrogen functionalities on the surface of the activated carbons and finally, the effects of these oxygen and nitrogen groups on the electrochemical performances of the corresponding electrodes have been comparatively studied and discussed, showing these materials as excellent candidates to form part of applicable supercapacitors.

## 2. Materials and Methods

### 2.1. Synthesis of Modified Activated Carbons Samples

The activated carbons (ACs) were prepared by chemical activation with KOH of three different woods: custard apple, fig tree, and olive tree following the method described previously [11]. The samples were prepared by impregnation with KOH in a weight ratio of 1:1. The mixtures were heated at 60 °C to dryness and after that at 110 °C until evaporation total of the water. The solids produced were carbonized under N_2_ flow (300 cm^3^ min^−1^) and heating rate of 5 °C min^−1^, at 300 °C for 1 h followed by activation at 800 °C for 2 h. The produced activated carbons were washed with HCl (1 M) and with distillated water until neutralization of the washing water and total elimination of chloride. The samples were designated as CK, FK, and OK, indicating that they were obtained from custard apple, fig tree, and olive tree, respectively.

The pre-prepared samples were treated with two oxidative agents for the introduction of oxygen surface groups: nitric acid and ammonium persulfate; and with ammonium carbamate and melamine for the introduction of nitrogen groups.

In details, the treatment with nitric acid was carried with 1 M diluted nitric acid at boiling temperature. Nitric acid (1 M) was slowly added through the funnel. The oxidation was carried out at the boiling temperature for 2 h. The oxidized ACs were washed with distillated water until the absence of nitrates, and then dried overnight at 50 °C. The prepared samples were denoted FKN, OKN, and CKN.

The treatment with ammonium persulfate (NH_4_)_2_S_2_O_8_ to introduce surface oxygen functionalities was performed as reported by Moreno-Castilla [24]. In detail, the treatment was carried out with a saturated solution of this salt in H_2_SO_4_ 1 M (1 g of carbon/10 mL of solution) at 25 °C for 48 h. After the treatment, the samples were washed with distilled water until absence of sulfates was reached. The prepared samples were denoted FKS, OKS, and CKS.

The treatment with melamine was prepared by mixing 0.5 g of activated carbon with 33 mg of melamine dissolved in 20 mL of ethanol as described in our previous work [11]. After stirring this slurry, the solvent was slowly removed by evaporation and the remaining residue was heat-treated at 750 °C for 1 h under N_2_ flow (60 cm^3^ min^−1^). The corresponding samples were denoted FKM, OKM, and CKM.

Finally, the introduction of nitrogen functionality by using ammonium carbamate was carried by mixing 2 g of ammonium carbamate and 2 g of activated carbon in 20 mL of distillated water. After stirring this slurry, the residue was heated at 550 °C for 1 h and 600 °C for 1 h, both under N_2_ flow (60 cm^3^ min^−1^). The prepared samples denoted FKC, OKC, and CKC.

### 2.2. Characterization

Textural characterization was carried out by gas adsorption, using N_2_ and CO_2_ at −196 °C and 0 °C, respectively, in a Quantachrome Autosorb-1 equipment (Anton Paar QuantaTec, Boynton Beach, FL, USA). The apparent surface area (S_BET_) together with: the micropore volume (W_0_), the mean micropore width (L_0_) and the microporous surface (S_mic_), were obtained applying the BET and Dubinin–Radushkevich equations, respectively. The total pore volume, V_total_, was considered as the volume of N_2_ adsorbed at P/P_0_ = 0.95 and the mesopore volume, V_meso_, was obtained by the difference between V_total_ and the micropore volume obtained from nitrogen adsorption.

The surface chemistry of the activated carbons was studied by X-ray photoelectron spectroscopy (XPS) and temperature programmed desorption coupled with mass spectrometry (TPD). TPD and XPS analysis were carried out as described elsewhere [11]. For TPD a heating rate of 20 °C min^−1^ to 1000 °C was used. Total oxygen content, O_TPD_, was measured counting the amount of CO and CO_2_ evolved [11]. During the XPS analysis C_1s_, O_1s_, N_1s_, and S_2p_ spectra were recorded and deconvoluted as described elsewhere [11]. 

### 2.3. Electrochemical Measurements

The electrochemical measurements were performed in a two electrodes system as described elsewhere [25,26]. The working temperature was 25 °C using H_2_SO_4_ (1 M) as electrolyte. Glass fibrous material was used as a separator. The preparation of the working electrodes was carried out as described elsewhere [26]. The voltage window was 0–0.9 V in H_2_SO_4_ (1 M). In the mentioned voltage interval, Cyclic voltammetry (CV) was performed at different scan rates (0.5, 2.5, 5, 10 and 20 mV s^−1^). The gravimetric capacitance obtained from CV, C_CV_ (F g^−1^), the gravimetric capacitance obtained from galvanostatic charge–discharge analyses, C_GD_ (F g^−1^), the capacitance value, C_max_, obtained from impedance spectroscopy measurements, and the electrical energies, E (Wh Kg^−1^) and power densities, P (W Kg^−1^) for two-electrode cell were calculated as described elsewhere [26]. Finally, the stability of supercapacitors was also monitored by charge–discharge cycles as described elsewhere [25].

## 3. Results and Discussion

### 3.1. Structural and Textural Characterization

Table 1 shows the characterization data obtained from N_2_ adsorption–desorption and CO_2_ adsorption isotherms; the results indicate a change after the modification with different treatments for the introduction of oxygen and nitrogen functionalities. Figure S1 collects the N_2_ adsorption–desorption isotherms at 77 K for CK-series as example.

From the data it can be seen that the treatment with melamine and ammonium carbamate, for all samples, increase the surface area and also the pore volume increase, which can be due to the thermal treatment at higher temperature and also the modification can cause the degradation of non-carbon impurities, resulting in an increase in surface area (CKC = 1706 m^2^ g^−1^, FKC = 1669 m^2^ g^−1^, OKC = 1314 m^2^ g^−1^). The only difference between the three samples was that the FK series shows a decreasing of the pore diameter; however, both CK and OK series show an increasing of the pore diameter. On the other hand, the results indicate a decreasing of micropore volume after the treatment with ammonium carbamate but a slow increasing with the melamine treatment. Furthermore, all samples show an increasing of the micropore diameter indicating a destruction, or fusion of microporous structure.

For all samples treated with melamine and ammonium carbamate, W_0_(N_2_) is inferior to W_0_(CO_2_) indicating the presence of constriction at micropores entrances and partial accessibility of N_2_ molecule at −196 °C. So, the treatment with melamine and ammonium carbamate for the introduction of nitrogen functionalities has the more significant effects on the microporous structure of all the three samples treated.

The results of the oxidation with the treatment with ammonium persulfate indicate decreasing in the surface area, mesoporous and microporous volume, and mesoporous and microporous diameter by partially destroying micro and mesopore walls. The oxidation with nitric acid indicates destruction of the pore structure and of mesopore and microporous volume. This is due to the destruction of pore walls and micropore blocking by oxygen-containing groups introduced during the chemical modification. The only exception is that the sample FKN shows lower pore destruction (0.11 cm^3^ g^−1^) than the both two other samples CKN (0.01 cm^3^ g^−1^) and OKN (0.01 cm^3^ g^−1^). Furthermore, of the three series of samples, all the samples with the same treatment have a similar value of the mesopore volume (0.09 cm^3^ g^−1^).

The surface chemistry of all activated carbon samples was characterized by XPS and TPD experiments. Table 2 contains the O and N surface contents determined by XPS as well as the quantification of the amount of CO and CO_2_ desorbed in these experiments. The results of the TPD indicate that samples have a wide range of surface oxygen groups being, in all the cases, the CO evolved groups larger than the CO_2_. The treatment of the three activated carbons CK, FK, and OK showed similar higher oxygen contents, CO/CO_2_ ratio. The samples treated with ammonium persulfate and nitric acid has a very higher amount of oxygen varying from 14.08 to 27.02%.

All the samples treated with nitric acid show lower O_XPS_ than O_TPD_ that is, there is a non-uniform oxygen surface groups distribution; on the contrary, ammonium persulfate treated samples present a uniform distribution of the oxygen content, O_XPS_ (CKS = 15.5 wt.%; FKS = 16.0 wt.%; OKS = 15.0 wt.%) are similar to O_TPD_ values (CKS = 14.0 wt.%; FKS = 15.1 wt.%; OKS = 14.4 wt.%). It is important to note that for oxidized samples, CO/CO_2_ ratio was generally lower than in those treated with melamine or ammonium carbamate because the oxidation mainly increased the amount of CO_2_-evolving groups such as carboxyl acid groups, which usually increases during oxidation treatments [24]. This increase in the oxygen surface functionalities is accompanied by a decrease in the hydrophobicity because oxygen functionalities with large polarity have been introduced, e.g., carboxyl groups. The different types of oxygen groups present on the surface of carbon materials decompose upon heating producing CO and CO_2_ at different temperatures. In this line, CO_2_ evolves at low temperatures as a consequence of the decomposition of the acidic groups, typically carboxylic groups and/or lactones [26]. However, the CO evolution takes place at higher temperatures and it is related to the decomposition of basic or neutral groups such as carbonyls, phenols, and ethers.

The treatment of the activated samples with melamine and carbamate ammonium increased the content on nitrogen, which was fixed forming part of pyridinic (N-6), pyrrolic, and/or pyridonic (N-5) and quaternary-N (N-Q) groups [23]. The treatment greatly increased the amount of N-6 functionalities and reduced the amount of N-Q functionalities. The results indicate that the melamine treatment fix more nitrogen contents than the ammonium carbamate and also that show higher oxygen contents in the surface than those treated with melamine.

In order to characterize the surface chemistry in the outermost layer of the materials, XPS analysis was performed. The deconvolution of C_1s_, O_1s_, N_1s_, and S_2p_ signals, and the corresponding peaks fitting showed the presence of diverse contributions to Binding Energies (BEs) that are displayed in Supplementary Figures S2–S4 and in Table 3, together with their corresponding percentages.

The C_1s_ spectrum for the all the modified samples contains two main peaks centered at 284.5 ± 0.1 (~63% on average) and 285.7 ± 0.2 eV (~18% on average) corresponding to C=C and C-C bonds, respectively [26,27,28]. Only minor peaks were detected at higher BE, which is typical for an activated carbon with a low oxygen content. It is important to note that after treatment with nitric acid and ammonium persulfate, samples that have been oxidized show an increase in the presence of O-C=O groups (288.6 ± 0.1 eV) [26,27,28] going from ~ 5% to ~ 11% on average.

The deconvolution of the spectra O_1s_ is presented in the Table 3, which can be deconvoluted into two peaks. The first one centered at 531.6 ± 0.4 eV attributed to the presence of the oxygen double bonded C=O groups. The second peak at 532.9 ± 0.5 eV indicates the presence of the singly bonded oxygen (-O-) in C-O [26,27,28]. An important observation is made when comparing the precursors used for the preparation of the activated carbons. For the samples prepared from custard apple and fig tree, the same distribution of oxygenated species is observed (40% of C=O groups and 60% of C-O groups, on average), however, for samples prepared from wood of olive tree this proportion changes (50% C=O and 50% C-O, on average)

The chemical state of nitrogen present in the treated samples with melamine and ammonium carbamate is further discussed on the basis of the XPS results. Deconvolution of the N_1s_ spectra resulted in four peaks. The first, centered at 398.3 ± 0.1 eV, designated N-6, is attributed to pyridinic-N, with the nitrogen atom in a six-membered ring and contributing with one p-electron to the aromatic π−system (~26% on average). The second peak at 399.4 eV can be attributed to the presence of nitrile groups present in the samples (~32% on average). The third peak centered at 400.5 eV, which is ascribed to pyrrolic-N or pyridone-N and will be referred as N-5 (~28% on average). Both groups have similar chemical environment for the nitrogen atom, with two p-electrons contributing to the π−system. Finally, the fourth peak at 401.6 ± 0.3 eV is attributed to quaternary nitrogen (N-Q) (~14% on average), which compared to pyridinic-N is defined as relatively more positively charge nitrogen incorporated in a graphene layer [29,30,31,32]. All samples showed a uniform distribution of the nitrogen groups introduced.

As expected, the samples treated with oxidizing agents showed a different distribution of these groups. For the samples oxidized with nitric acid, two notable changes are observed in comparison with the aforementioned samples: a) the peak attributed to pyridinic-N groups close to 398.3 eV is not appreciable; and b) the presence of a broad peak centered at 405.6 eV is observed (~42% on average), which can be attributed to nitrogen of the NO_2_ groups [33]. It is important to highlight that for the samples treated with ammonium persulfate, the presence of nitrogen groups in not detectable by means of XP spectroscopy analysis.

Finally, only for the samples treated with ammonium persulfate a small amount of sulphur was detected on the surface due to the epoxy groups formed during the synthesis of the materials. The S_2p_ peak could be deconvoluted with a doublet of sulfate (S2p_3/2_ at 168.2 eV and S2p_1/2_ at 169.4 eV) indicating the presence of groups SO_2_ in the surface of these materials [34].

### 3.2. Electrochemical Characterization

**Figure 1:**
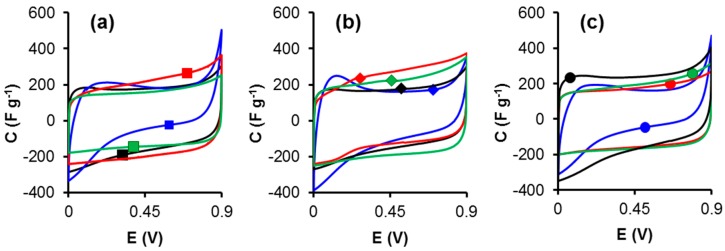
Cyclic voltammograms at 0.5 mVs^−1^ of all samples: (**a**) CK-series, (**b**) FK-series and (**c**) OK-series in H_2_SO_4_ 1 M. Treatments: melamine (red), ammonium carbamate (green), nitric acid (blue), and ammonium persulfate (black).

The electrochemical characterization was evaluated in H_2_SO_4_ 1 M with two electrode system. Figure 1 shows the CV curves of all the electrodes tested between 0 and 0.9 V at the scan rate of 0.5 mV s^−1^. It can be seen that the samples treated with melamine, ammonium carbamate, and ammonium persulfate, show a quasi-rectangular shape, which is a feature of electrochemical double-layer capacitors. In contrast, the samples treated with nitric acid show non-rectangular shape due to the effect of the presence of the higher content of oxygen surface groups evolved as CO_2_ which induces some diffusional restrictions due to the interaction between strong oxygen surface groups and the ions of the electrolyte, setting aside the CVs curves from the purely capacitive shape.

**Figure 2:**
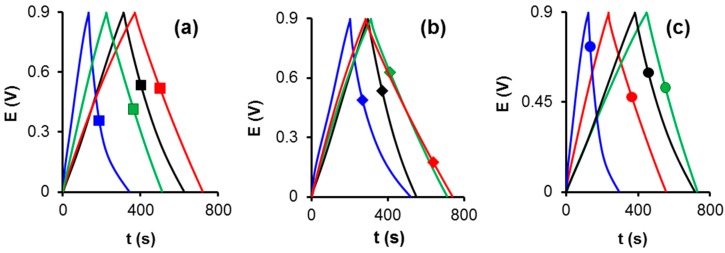
Galvanostatic charge-discharge curves at 125 mA g^−1^ for all samples: (**a**) CK-series, (**b**) FK-series and (**c**) OK-series. Treatments: melamine (red), ammonium carbamate (green), nitric acid (blue), and ammonium persulfate (black).

Figure 2 shows the galvanostatic charge–discharge curves, it is clear that the curves of all oxidized samples exhibit a slightly distorted triangular shape, due to the pseudo-capacitive behavior of the oxygen functional groups. This distortion was more significant for the nitric acid treated samples. In contrary, the nitrogen doped samples show a symmetric triangular shape indicating a good diffusion inside the pore structure.

**Figure 3:**
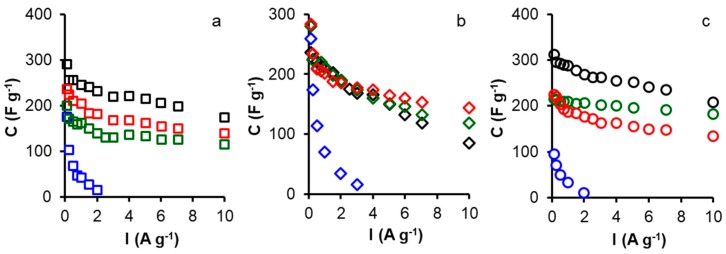
Variation of the specific capacitance with current density in H_2_SO_4_ 1 M for all samples: (**a**) CK-series, (**b**) FK-series, and (**c**) OK-series. Treatments: melamine (red), ammonium carbamate (green), nitric acid (blue), and ammonium persulfate (black).

The specific capacitance versus current density for all samples was further collected to study the rate performance. The results are presented in Figure 3 and indicate that all the treatment process give samples with a good stability at higher current density of 10 A g^−1^ except for samples treated with nitric acid, which present a lower stability and a fast decreasing of the capacitance with the increasing of the current density. On the other hand, it is found that the samples oxidized by ammonium persulfate show the largest specific capacitances at current densities between 250 mA g^−1^ and 10 A g^−1^ for all modified samples except for FK series at high current densities. The maximum specific capacitance of 312 F g^−1^ was attained at a current density of 125 mA g^−1^ for OKS, which is much higher than those of FKS (237 F g^−1^) and CKS (290 F g^−1^).

Remarkably, the specific capacitance of ammonium persulfate treated samples cannot still be maintained at higher current density, which is due to the effect of oxygen functionalities. The retention ratio of those samples from 125 mA g^−1^ to 10 A g^−1^ was between 36% and 67%. These above results clearly show and confirm that the introduction of a large amount of oxygen-containing groups and the increased amount of mesopores are very effective enhancing the electrolyte accessibility, leading to fast ion response and higher capacitance and that the presence of surface quinone groups increases the capacitance of oxidized activated carbons by introducing pseudo-capacitance effects; nevertheless, the oxidation of the surface no always produced one way effects because also fixes carboxyl groups which thereby increasing its ohmic resistance [35] due to their high polarity, bind water molecules that hinder and retard electrolyte diffusion into the microporosity.

The nitric acid treated samples show a fast decreasing in the capacitance at higher current densities due to the destruction of the pore structure and also to the higher values of oxygen present in the surface functionalities of treated samples.

It is known that the increase in the population of the CO-desorbing complexes can has positive effect on the capacitance, while the CO_2_-desorbing complexes show a negative effect in double-layer formation. Upon heat-treating the oxidized carbon, most of the CO_2_-desorbing complexes were removed while the population of CO-desorbing complexes reached a maximum. This treatment has produced electrodes with the highest capacitance. Cyclic voltammetry showed that the presence of the CO desorbing complexes significantly enhanced the double-layer formation and thus the capacitance. This indicates that due to the local changes of electronic charge density a proton adsorbed by a carbonyl or quinone-type site facilitates an excess specific double-layer capacitance. The faradic current increased with the total number of oxygen atoms on the surface, indicating that both the CO- and CO_2_-desorbing complexes enhanced the redox process.

It has been reported that surface N functionalities are electrochemically active because they are electron-rich [36,37]. In this way, protons can be attracted to the electrode surface, producing pseudo-capacitive interactions [38]. For the melamine nitrogen doped samples, the result indicate that the sample FKM presents the higher capacitance 283 F g^−1^ followed by CKM 236 F g^−1^ and OKM 225 F g^−1^, this difference of the capacitance can be explained by the nitrogen content of all the samples (Table 3) which indicates that the samples FKM has more than 2% of nitrogen contrary to both the two others ones which have a nitrogen content of 1.6% for CKM and 1% for OKM. This can also be explained by the difference of surface area between all samples. The results (Table 2) indicate that those samples show good retention stability between 50 and 60% due to an adequate pore structure of all samples facilitating a good penetration of the electrolyte inside the porosity at high current densities.

For the ammonium carbamate modified samples, all the samples present the seam values of the nitrogen content 0.8%, so the difference of the capacitance and the electrochemical performance can be explained by the difference of the porous structure. The results (Table 4) indicate that the sample FKC has the higher capacitance of 280 F g^−1^ compared to both of the two other samples CKC 199 F g^−1^ and OKC 216 F g^−1^. This difference can be explained by the difference in pore volume and especially the micropores volume. The sample FKC has a micropore volume of 0.67 cm^3^ g^−1^ which is higher than both the other ones. Those samples present capacitance retention at current density of 10 A g^−1^ varying from 42% for FKC to 84% for OKC and 57% for CKC. This difference is due in one part to the pore diameter and also to the difference of oxygen amount. It is known that the presence of oxygen in the surface chemistry can affect the capacitance retention and especially at higher current densities. This remarkable electro-chemical performance, as the high specific capacitances can be due to the excellent and adequate textural characteristics which makes possible a large adsorption interface for electrolyte to form the electrical double layer, leading to a great electrochemical double layer capacitance; but also the doping with hetero-atoms enhances the surface interaction of these materials with the aqueous electrolyte, increasing the accessibility of electrolyte ions. The hetero-atoms groups can also provide considerable pseudo-capacitance improving the overall capacitance.

**Figure 4:**
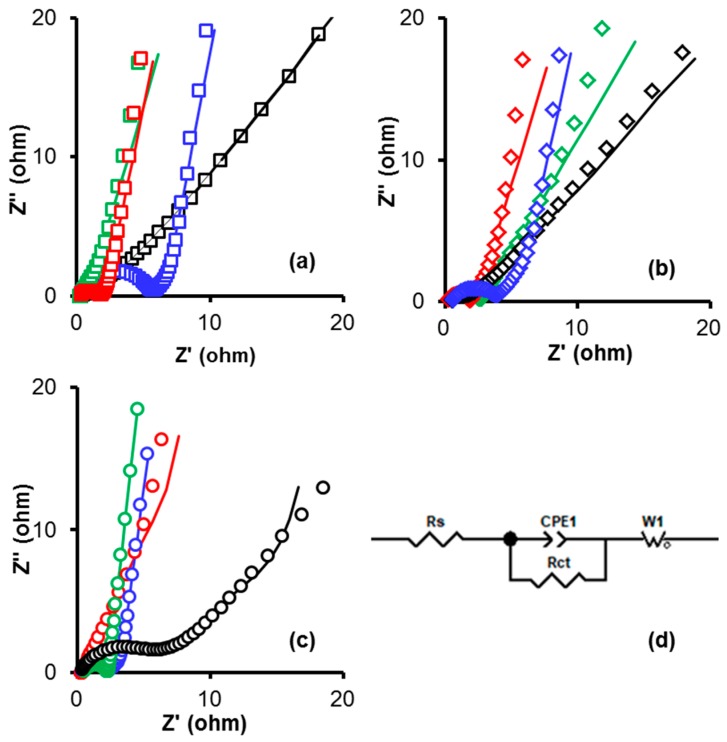
Nyquist plots obtained from EIS experiments on H_2_SO_4_ 1 M for all the samples: (**a**) CK-series, (**b**) FK-series, (**c**) OK-series, and (**d**) equivalent circuit model. Treatments: melamine (red), ammonium carbamate (green), nitric acid (blue), and ammonium persulfate (black).

In order to get insights on the influence of surface chemistry on the electrochemical performance of the electrodes, electrochemical impedance spectroscopy (EIS) was used. The Nyquist plots and their corresponding equivalent circuits are shown in Figure 4 (the continuous line represents the adjustment of the experimental data to the equivalent circuit model). In order to obtain kinetic parameters, impedance data were fitting to the equivalent circuit proposed by Zhang et al. [39] using ZVIEW software, version 2.7, the results are shown in Table 5. The equivalent circuit proposed is based in a fractional-order model, which consists of a series resistor (R_s_), a parallel resistor (R_ct_), a Constant-Phase Element (CPE), and a Walburg-like element (W). The ionic resistance of the electrolyte, the intrinsic resistance of the active material, and the contact resistance at the electrode/current collector interface are contained in the R_s_. Therefore an R_ct_ represents the faradic charge transfer resistance at the interface between the current collector and the active material. Finally, the W represents the diffusive resistance.

To evaluate the goodness of fit of the experimental data to the equivalent circuit, the statistic X_i_^2^ was used. We obtained values in the range of 1.0 × 10^−3^ for all of the fitted data (Table 5). Thus, we determined that the proposed equivalent circuit fits the experimental data reasonably well.

Once oxygen and nitrogen have been introduced to the material, R_s_ decreases dramatically, so that the formation is no longer perceptible in the figure, indicating a decrease in the formation of the solid-electrolyte interface layer. For all the samples treated with melamine the R_s_ value is the lowest (0.2 Ω), so that this effect can be attributed to a decrease in the formation of the solid-electrolyte interface layer due to the increase in the nitrogen functional groups which improve the hydrophobicity of the material, thus making the surface more wettable with electrolyte [40,41].

The charge transfer resistances (R_ct_) of all nitrogen treated samples are varying between 0.05–2.18 Ω. A faster ion diffusion and lower impedance on the electrode/electrolyte are taking place, being this deduced from the small semicircles formed when the faradic charge transfer resistance (R_ct_) of electrodes in H_2_SO_4_ electrolyte is represented. Those lower resistances due to the lower change in the pore structure after the melamine and ammonium carbamate treatments. In contrast, the oxygenated samples show a higher internal resistance compared to nitrogenated ones (for example: OKM = 0.05 Ω, OKC = 1.74 Ω vs. OKN = 5.04 Ω, OKS = 2.00 Ω). This higher resistance due to the lower pore diameter resultants from the treatment with nitric acid and ammonium persulfate. In addition, all curves show a Walburg-like element angle higher than 45°, indicating the suitability of the electrode materials for supercapacitors.

The relaxation time constant (τ) is a quantitative measure of the speed with which the device can be discharged and this can be calculated using the equation τ = 1/(2 f_0_), being f_0_ the transition frequency between a pure capacitive and a pure resistive behavior that can be obtained from the maximum within the variation of the imaginary part of the capacitance (C”) against the frequency. Results collected in Table 5 show that nitrogen doped samples present the faster discharging time (1.3 s on average) compared to the oxygenated ones and that the acid nitric treated samples (61.97 s on average) due to the lower microporosity.

**Figure 5:**
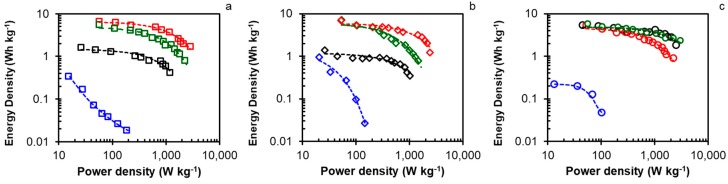
Ragone plots on H_2_SO_4_ 1 M for all samples: (**a**) CK-series, (**b**) FK-series, and (**c**) OK-series. Treatments: melamine (red), ammonium carbamate (green), nitric acid (blue), and ammonium persulfate (black).

The Ragone plots of all the electrodes tested are displayed in Figure 5 and the maximum and minimum energies and power densities are shown in Table 6. When analyzing the results, it is clear that both the maximum energy density (E_max_) and the maximum power density (P_max_) have been considerably improved with the introduction of nitrogen groups on the surface, since the series of materials treated with melamine and sodium carbamate show better results (average E_max_ = 6.25 Wh kg^−1^; average P_max_, = 2363 W kg^−1^) than the oxidized samples (average E_max_ = 1.89 Wh kg^−1^; average P_max_, = 854 W kg^−1^). Additionally, these results are adequately related to the textural properties of the materials, since alkaline treatments increase the volume of mesopores. Since the power density is influenced by the transport of internal pore ions, this behavior can be explained to be due to the conditions of the porosity channels and the hydrophobicity of the surface.

**Figure 6:**
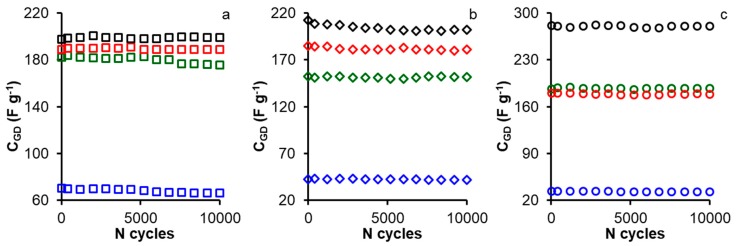
Variation of the gravimetric capacitance (C_GD_) with the number of charge discharge cycles at 1 A g^−1^ in the potential window between 0 and 0.9 V in H_2_SO_4_ 1 M for all samples: (**a**) CK-series, (**b**) FK-series, and (**c**) OK-series. Treatments: melamine (red), ammonium carbamate (green), nitric acid (blue), and ammonium persulfate (black).

The energy released decreased at higher power density; but the energy densities of nitrogen doped samples were acceptable at higher power density. The long-term stability of electrodes is a very important property that can limit the application of any materials as supercapacitors for practical applications. Figure 6 shows the variation in the gravimetric capacitance with the number of charge–discharge cycles at a constant current density of 1 A g^−1^ employing H_2_SO_4_ 1 M as electrolyte. After 10,000 cycles the retention capacity for the all modified samples are between 97.0%, and 100%.

## 4. Conclusions

In this study, three series of ACs were prepared by KOH activation of different woods: Custard apple tree (CK-series), Fig tree (FK-series) and Olive tree (OK-series). The ACs were treated with four agents: melamine, ammonium carbamate, nitric acid, and ammonium persulfate, for the introduction of nitrogen and oxygen groups on the surface of materials with the aim to study the influence of surface chemistry on the electrochemical performance of biomass-derived carbon electrodes for its use as supercapacitors. The results showed that the treatments introduce different nitrogen functionalities, such as pyridine quaternary-N and oxidized nitrogen, which improve the wettability and the ions transfers. The effectiveness of the activation and doping methods is very good, obtaining comparable materials in porosity and relative chemical properties, in spite of the different origins of the woods. The obtained electro-chemical results are also very remarkable, since after 10,000 cycles, the retention capacity for the all modified samples are between 97.0% and 100%.; with the advantage that very cheap waste materials can be used for the supercapacitor development. Nevertheless, it should be clarified that the treatment with nitric acid, although it is also very reproducible in its effects, is not an advisable doping treatment because it destroys the microporosity and, therefore, reduces the electrochemical performance. Finally, the high electro-chemical performance, such as the very remarkable specific capacitances, can be probably due to the excellent and adequate textural characteristics, as high surface areas, which makes possible a large adsorption interface for electrolyte to form the electrical double layer, leading to a great electrochemical double layer capacitance; as well as the doping with hetero-atoms which enhances the surface interaction of these materials with the aqueous electrolyte, increasing the accessibility of electrolyte ions.

## Figures and Tables

**Table 1:** Textural characteristics of modified activated carbons.

Sample	N_2_					CO_2_	
	S_BET_ m^2^/g	W_0_(N_2_) cm^3^/g	L_0_(N_2_) nm	V_total_ cm^3^/g	V_mes_ cm^3^/g	W_0_(CO_2_) cm^3^/g	L_0_(CO_2_) nm
CK	1504	0.59	1.20	---	---	0.35	0.70
CKM	1525	0.60	1.16	0.75	0.15	0.37	0.70
CKC	1706	0.68	1.27	0.85	0.17	0.06	0.95
CKN	46	0.01	4.84	0.10	0.09	0.13	0.46
CKS	1042	0.41	1.16	0.54	0.13	0.32	0.61
FK	1024	0.41	1.30	---	---	0.31	0.70
FKM	1575	0.63	1.26	0.80	0.17	0.35	0.67
FKC	1669	0.67	1.28	0.84	0.17	0.06	0.99
FKN	287	0.11	1.40	0.20	0.09	0.07	0.93
FKS	1114	0.44	1.36	0.59	0.15	0.32	0.62
OK	1273	0.49	1.30	---	---	0.34	0.70
OKM	1400	0.56	1.34	0.77	0.21	0.23	0.63
OKC	1314	0.53	1.32	0.69	0.16	0.30	0.71
OKN	153	0.06	2.81	0.15	0.09	0.14	0.47
OKS	1075	0.42	1.30	0.58	0.16	0.25	0.61

**Table 2:** Surface chemistry of the modified activated carbons.

Sample	O_xps_ (wt.%)	N_xps_ (wt.%)	O_TPD_ (wt.%)	CO (mmol g^−1^)	CO_2_ (mmol g^−1^)	CO/CO_2_
CKM	1.7	1.6	1.0	0.11	0.26	0.42
CKC	3.7	0.8	2.1	0.45	0.43	1.05
CKN	21.4	1.1	25.5	1.16	7.41	0.16
CKS	15.5	0.9	14.0	0.87	3.96	0.22
FKM	1.9	2.1	1.0	0.19	0.23	0.83
FKC	4.3	0.7	2.1	0.48	0.42	1.14
FKN	20.6	0.9	25.3	1.22	7.32	0.17
FKS	16.0	0.5	15.1	0.94	4.26	0.22
OKM	6.1	1.0	3.7	0.26	1.03	0.25
OKC	5.7	0.9	7.0	0.41	1.98	0.21
OKN	22.0	1.4	27.0	1.78	7.85	0.23
OKS	15.0	0.2	14.4	0.89	4.02	0.22

**Table 3:** XPS data obtained after deconvolution of the high-resolution XP spectra.

Sample	C_1s_ (eV)	FWHM (eV)	Peak (%)	O_1s_ (eV)	Peak (%)	N_1S_ (eV)	Peak (%)	S2p_3/2_ (eV)	Peak (%)
CKM	284.5	1.34	65	531.6	33	398.3	33		
	285.7		17	532.9	67	399.4	28		
	286.9		7			400.5	24		
	288.4		5			401.6	15		
	290.2		4						
	291.7		2						
CKC	284.5	1.48	65	531.3	49	398.4	20		
	285.8		18	533.4	51	399.4	31		
	287.2		7			400.5	31		
	288.5		5			401.6	18		
	290.3		4						
	291.6		1						
CKN	284.5	1.45	60	531.4	39	399.3	16		
	285.8		19	533.0	61	400.5	11		
	286.9		6			401.5	34		
	288.6		12			405.5	40		
	290.1		3						
	291.5		1						
CKS	284.6	1.40	61	531.5	36			168.2	62
	285.8		19	533.0	64			169.5	38
	287.0		6						
	288.5		11						
	290.3		3						
	291.7		1						
FKM	284.6	1.35	62	531.4	36	398.4	28		
	285.6		19	533.0	64	399.4	31		
	286.8		8			400.5	28		
	288.3		5			401.9	13		
	290.2		4						
	291.6		1						
FKC	284.6	1.35	65	531.1	36	398.4	28		
	285.8		18	533.3	64	399.4	31		
	287.1		7			400.5	28		
	288.5		5			401.9	13		
	290.3		4						
	291.5		1						
FKN	284.6	1.43	58	531.5	36	399.6	12		
	285.8		21	533.1	64	400.5	13		
	286.9		5			401.6	35		
	288.6		12			405.7	40		
	290.1		2						
	291.5		1						
FKS	284.5	1.43	62	531.6	40			168.2	67
	285.9		16	533.0	60			169.4	33
	287.0		7						
	288.5		11						
	290.3		4						
	291.9		1						
OKM	284.6	1.37	66	531.4	53	398.3	27		
	285.7		17	533.0	47	399.4	31		
	286.9		7			400.5	27		
	288.5		4			401.6	16		
	290.1		4						
	291.5		2						
OKC	284.5	1.38	66	531.2	48	398.3	20		
	285.8		17	533.0	52	399.5	40		
	287.1		6			400.5	32		
	288.6		5			401.9	8		
	290.2		4						
	291.6		1						
OKN	284.6	1.45	58	531.5	39	399.5	16		
	285.7		21	533.1	61	400.5	14		
	287.0		6			401.6	26		
	288.6		12			405.6	45		
	290.1		2						
	291.3		1						
OKS	284.5	1.42	62	531.5	48			168.2	71
	285.9		18	533.1	52			169.2	29
	287.0		7						
	288.5		9						
	290.2		4						
	291.6		1						

**Table 4:** Electrochemical capacitances (F g^−1^) of modified samples in H_2_SO_4_ 1 M. Retention capacitance at 10 A g^−1^ referred to 125 mA g^−1^.

Sample	Cv 0.5 mV s^−1^	Ccp 125 mA g^−1^	Ccp 2 A g^−1^	Ccp 10 mA g^−1^	Retention (%)
CKM	205	236	181	139	59
CKC	153	199	139	115	58
CKN	153	176	16	-	8*
CKS	180	290	231	174	60
FKM	200	283	186	144	51
FKC	210	280	190	119	43
FKN	169	259	35	-	14*
FKS	171	237	189	85	36
OKM	167	225	176	135	60
OKC	180	216	206	183	85
OKN	143	145	11	-	48*
OKS	218	312	268	208	67

* Retention capacitance at 2 A g^−1^.

**Table 5:** Equivalent series resistance (R_s_), charge transfer resistance (R_CT_), and C_max_ at 1 mHz, from EIS.

Sample	R_s_ (Ω)	R_ct_ (Ω)	X_i_^2^	τ (s)	C’max (F g^−1^)
CKM	0.20	1.45	2.1 × 10^−3^	0.62	170
CKC	0.21	1.32	3.1 × 10^−3^	0.62	134
CKN	0.30	4.45	5.0 × 10^−3^	159.17	196
CKS	0.24	5.18	9.0 × 10^−4^	1.98	118
FKM	0.25	1.48	3.7 × 10^−3^	1.11	171
FKC	0.51	2.18	1.7 × 10^−3^	3.55	177
FKN	0.42	1.40	1.5 × 10^−3^	49.41	173
FKS	0.55	2.94	1.7 × 10^−3^	1.48	166
OKM	0.29	0.05	1.4 × 10^−3^	1.48	141
OKC	0.48	1.74	4.0 × 10^−4^	0.46	157
OKN	0.38	5.04	3.9 × 10^−3^	159.17	134
OKS	0.22	2.00	9.0 × 10^−4^	0.62	141

**Table 6:** Maximum and minimum energy densities (Wh Kg^−1^) and power densities (W Kg^−1^) of all samples from Ragone’s plots.

Sample	P_max_ W kg^−1^	E_min_ Wh kg^−1^	E_max_ Wh kg^−1^	P_min_ W kg^−1^
CKM	2825	1.72	6.61	57
CKC	2245	0.81	5.57	57
CKN	184	0.02	0.35	15
CKS	1180	0.42	1.64	26
FKM	2396	1.23	6.90	53
FKC	1461	0.79	7.10	54
FKN	147	0.03	0.95	21
FKS	997	0.12	1.40	26
OKM	2214	0.92	5.44	53
OKC	3039	2.35	5.88	56
OKN	111	0.01	0.22	13
OKS	2506	1.82	5.41	45

## Supplementary Materials

The following are available online at https://www.mdpi.com/1996-1944/12/15/2458/s1, Figure S1. N_2_ adsorption and desorption isotherms at 77K of CK-series samples. Figure S2: High resolution XPS deconvoluted spectra in the corresponding regions: (a) C_1s_, (b) O_1s_, (c) N_1s_ and (d) S2p_3/2_ for the activated carbons prepared from Custard apple tree wood (CK-Serie). Figure S3: High resolution XPS deconvoluted spectra in the corresponding regions: (a) C_1s_, (b) O_1s_, (c) N_1s_ and (d) S2p_3/2_ for the activated carbons prepared from Fig tree wood (FK-Serie). Figure S4: High resolution XPS deconvoluted spectra in the corresponding regions: (a) C_1s_, (b) O_1s_, (c) N_1s_ and (d) S2p_3/2_ for the activated carbons prepared from Olive tree wood (OK-Serie).
