# Supplementary material for: Influence of Surface Chemistry on the Electrochemical Performance of Biomass-Derived Carbon Electrodes for its Use as Supercapacitors
Source: Materials (Basel). 2019 Aug 2;12(15):2458. doi: 10.3390/ma12152458 (PMC6696370; doi:10.3390/ma12152458)
Supplement: Supplementary file 1 [file materials-12-02458-s001.pdf]

materials

SUPPLEMENTARY MATERIAL

Influence of Surface Chemistry on the Electrochemical Performance of Biomass-Derived Carbon Electrodes for its Use as Supercapacitors

Abdelhakim Elmouwahidi [1], Esther Bailón-García [1], Luis A. Romero-Cano [2], Ana I. Zárate-Guzmán [3], Agustín F. Pérez-Cadenas [1,*] and Francisco Carrasco-Marín [1]

1 Research Group in Carbon Materials, Inorganic Chemistry Department, Faculty of Sciences, University of Granada, Campus Fuente Nueva s/n. 18071 Granada, Spain
2 Facultad de Ciencias Químicas, Universidad Autónoma de Guadalajara, Av. Patria 1201, Zapopan, Jalisco C. P. 45129, México
3 Centro de Investigación y Desarrollo Tecnológico en Electroquímica (CIDETEQ) S.C., Parque Tecnológico Sanfandila, Pedro Escobedo, Querétaro 760703, México
* Correspondence: afperez@ugr.es; Tel.: +34 958243316

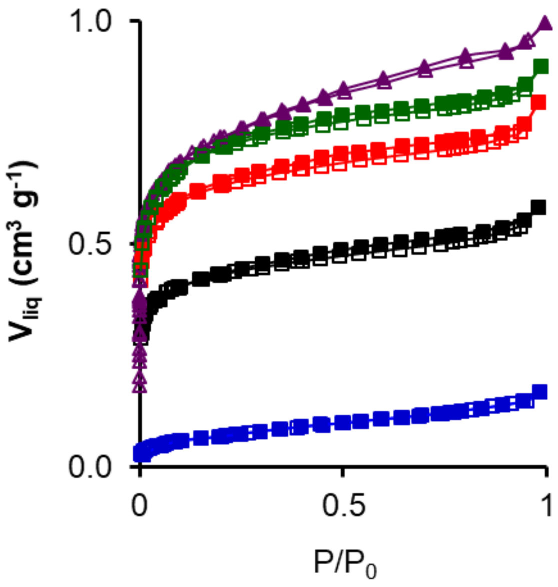

Figure S1. N_2 adsorption (open symbols) and desorption (close symbols) isotherms at 77K of CK-series samples. Sample CK is in purple. Treatments: melamine (red), ammonium carbamate (green), nitric acid (blue) and ammonium persulfate (black).

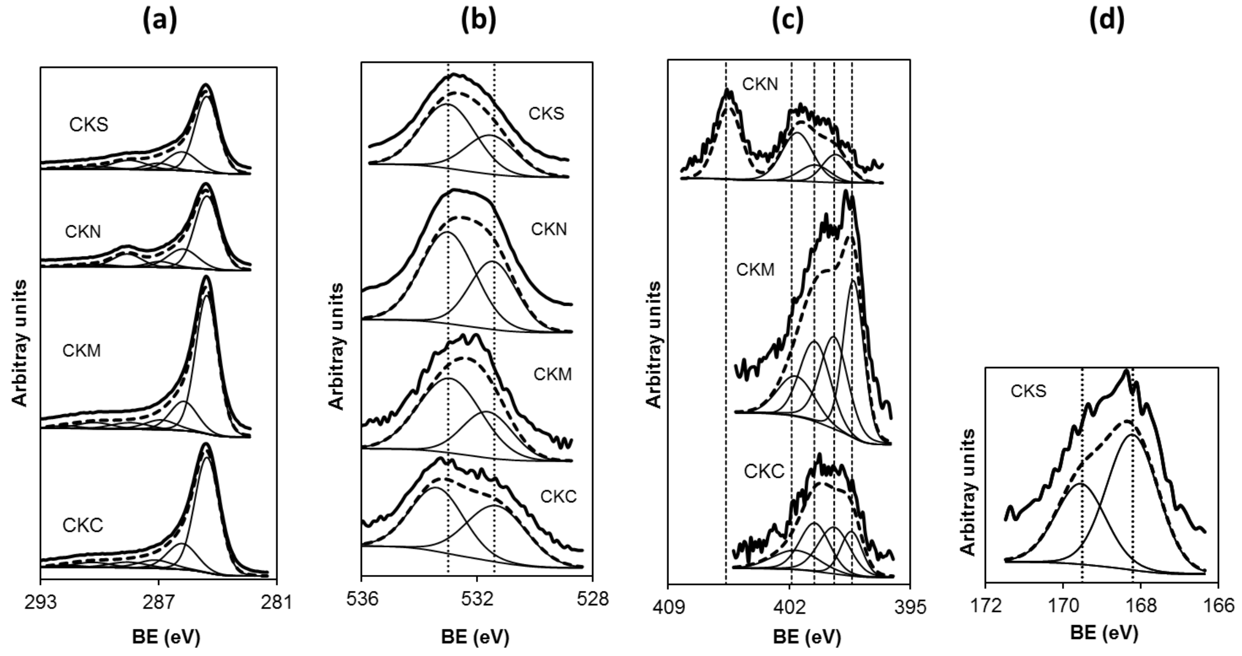

Figure S2. High resolution XPS deconvoluted spectra in the corresponding regions: (a) C_{1s}, (b) O_{1s}, (c) N_{1s} and (d) $S2p_{3/2}$ for the activated carbons prepared from Custard apple tree wood (CK-Serie).

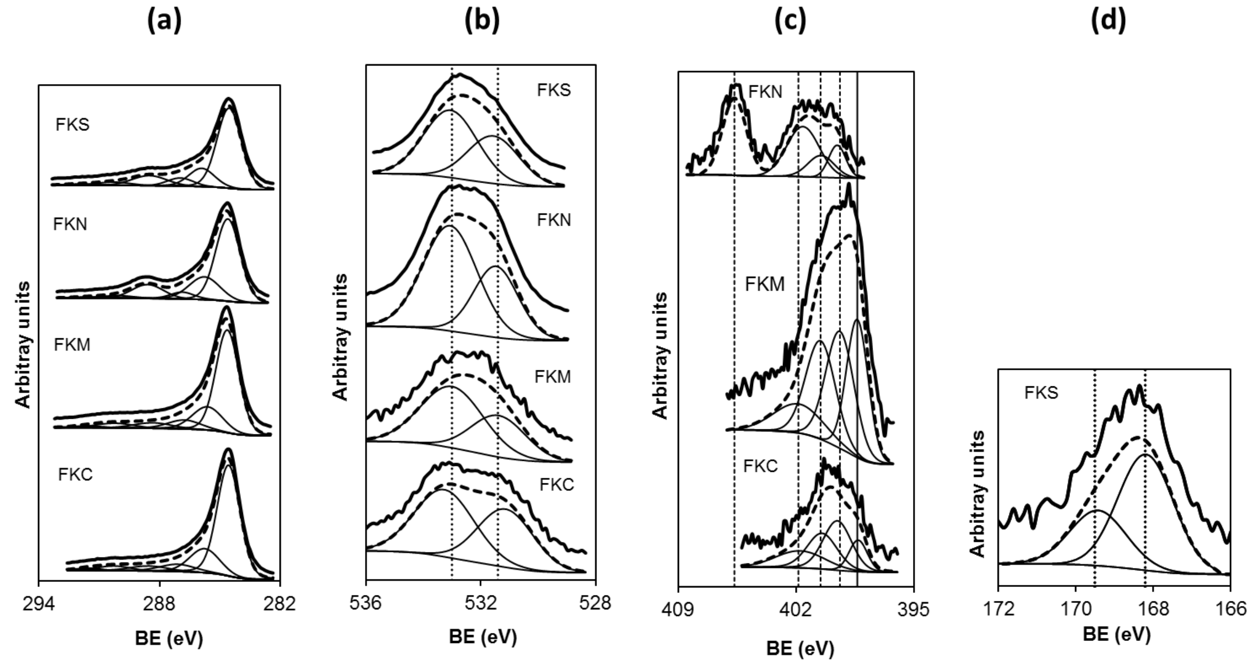

Figure S3. High resolution XPS deconvoluted spectra in the corresponding regions: (a) C_{1s}, (b) O_{1s}, (c) N_{1s} and (d) $S2p_{3/2}$ for the activated carbons prepared from Fig tree wood (FK-Serie).

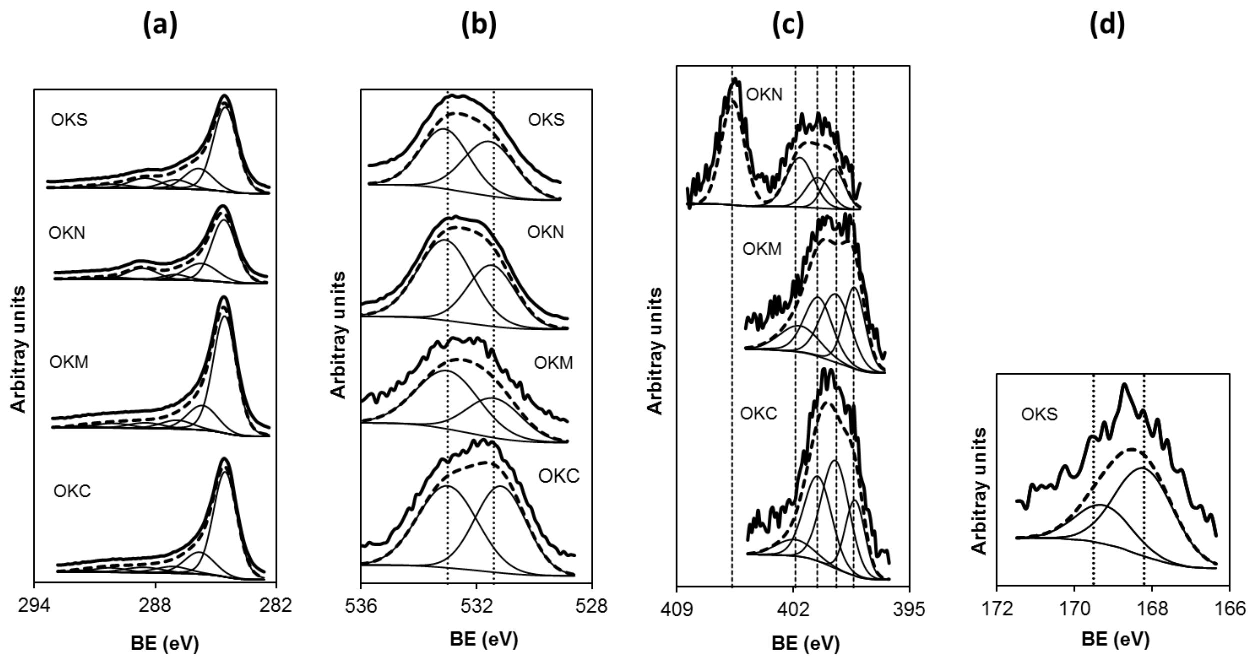

Figure S4. High resolution XPS deconvoluted spectra in the corresponding regions: (a) C_{1s}, (b) O_{1s}, (c) N1s and (d) $S_{2p3/2}$ for the activated carbons prepared from Olive tree wood (OK-Serie).
